# Liver dysfunction as a rare presentation of ICD lead-induced tricuspid regurgitation: a case report

**DOI:** 10.1093/ehjcr/ytae600

**Published:** 2024-11-13

**Authors:** Suzannah Fleming, Konstantinos Somarakis, Gareth Squire, Jonathan Goldney, Ian Loke

**Affiliations:** Cardiology Department, University Hospitals of Leicester NHS Trust and University of Leicester, Gwendolen Rd, Leicester LE5 4PW, UK; Cardiology Department, University Hospitals of Leicester NHS Trust and University of Leicester, Gwendolen Rd, Leicester LE5 4PW, UK; Cardiology Department, University Hospitals of Leicester NHS Trust and University of Leicester, Gwendolen Rd, Leicester LE5 4PW, UK; Cardiology Department, University Hospitals of Leicester NHS Trust and University of Leicester, Gwendolen Rd, Leicester LE5 4PW, UK; Diabetes Research Centre, Diabetes Research Centre, College of Life Sciences, University of Leicester, Gwendolen Rd, Leicester LE5 4PW, UK; Cardiology Department, University Hospitals of Leicester NHS Trust and University of Leicester, Gwendolen Rd, Leicester LE5 4PW, UK

**Keywords:** Implantable cardiac defibrillator, Case report, Congestive hepatopathy, Tricuspid regurgitation

## Abstract

**Background:**

Implanted cardiac devices with right ventricular leads can cause tricuspid regurgitation and subsequent heart failure. In these patients, congestive hepatopathy because of tricuspid regurgitation is well documented; however, the presentation of liver dysfunction without overt heart failure is rare.

**Case summary:**

We report a case of a 56-year-old man with presumed hypertrophic cardiomyopathy, presenting with jaundice without signs of decompensated heart failure, 8 years post-implantation of dual-chamber implantable cardiac defibrillator (ICD). A workup for abnormal liver function, including a liver biopsy, determined congestive hepatopathy as the cause. Cardiac imaging revealed severe tricuspid regurgitation caused by right ventricular ICD lead causing tricuspid valve malposition. Management included both tricuspid valve replacement and extraction of ICD, and there was an excellent recovery.

**Discussion:**

This is a rare case where severe tricuspid regurgitation and congestive hepatopathy secondary to an ICD lead presented with jaundice in the absence of signs of decompensated heart failure. In patients with an implanted cardiac device presenting with signs of liver dysfunction, congestive hepatopathy secondary to tricuspid regurgitation should be considered.

**Learning points**

- Liver dysfunction associated with severe tricuspid regurgitation is a potential complication of implantable cardiac devices with transvenous right ventricular (RV) lead.
- Tricuspid regurgitation due to a transvenous RV lead can cause isolated congestive hepatopathy, even in the absence of other heart failure signs. Prompt investigation and intervention are required in such patients.
- Tricuspid annuloplasty or tricuspid valve replacement, with explantation of implanted cardiac device, led to improvement of liver dysfunction and was a suitable therapeutic intervention in our case.

## Introduction

Implantable cardiac defibrillators (ICDs) were first implanted in humans in the 1980s.^1^ Now, over 200 000 ICDs are implanted annually worldwide, with over 4000 implanted per year in England.^2^

In patients at high risk of serious ventricular arrhythmia, implantation of ICD aims to prevent sudden cardiac death.^1^ Primary prevention indications include both patients with heart failure with reduced left ventricular (LV) fraction of <35% and those with an inherited cardiac condition such as hypertrophic obstructive cardiomyopathy. Secondary prevention indications include those with a history of a life-threatening ventricular arrhythmia with a non-treatable cause.^3^

Complications of ICD include tricuspid regurgitation (TR), due to lead placement; infection; access-related complications; and procedure failure.^4^ The relationship between right ventricular (RV) leads causing tricuspid valve leaflet dysfunction with subsequently worsening or causing TR is well established.^5^ A retrospective cohort study found that the severity of TR was significantly worse in transvenous lead ICD vs. subcutaneous ICD (S-ICD), suggesting that the increased risk of TR in populations with transvenous lead ICD is not purely progression of TR secondary to underlying cardiac disease.^6^

Tricuspid regurgitation can cause elevated right heart pressures, which result in an increase in both venous and hepatic sinusoid pressures.^7^ This commonly causes clinical signs such as peripheral oedema and raised jugular venous pressure (JVP). The sustained increase in hepatic venous pressure (hepatic congestion) can lead to liver cirrhosis.^7^ To the best of our knowledge, a patient presenting with jaundice due to liver dysfunction secondary to TR caused by an implanted device with a RV lead, without overt signs of significant heart failure, has not been previously reported.

## Summary figure

**Figure:**
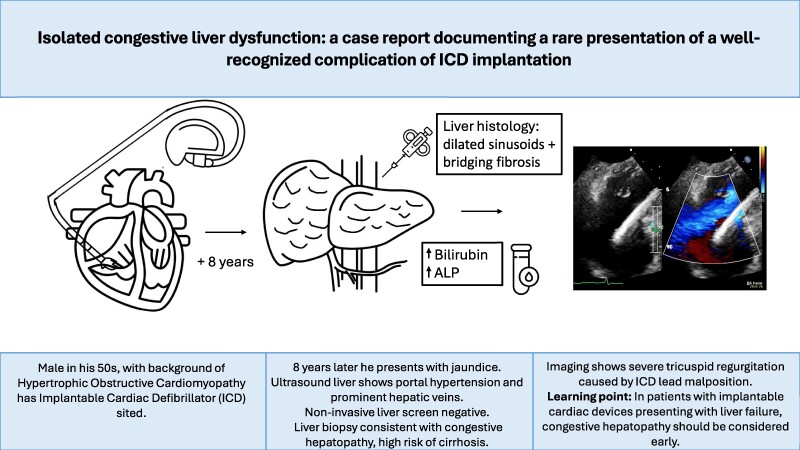


## Case presentation

A 56-year-old male had a dual-chamber ICD implanted due to unexplained syncope on a background of hypertrophic cardiomyopathy (HCM) and a family history of sudden cardiac death. His HCM sudden cardiac death risk score was 7.18%.^8^ He had a past medical history of hypertension and atrial fibrillation (AF). Seven years post-implantation, following diagnosis of AF, the device was downgraded to a single-chamber ICD with capped atrial lead. Following this, 8 years post-implantation, he presented with jaundice and pruritus without any symptoms or signs to suggest decompensated heart failure. He denied heavy alcohol intake and had no family history of liver disease. On examination, he was jaundiced, had an irregularly irregular pulse and normal heart sounds, and abdomen was soft with no liver tenderness, with no peripheral oedema, or other stigmata of liver failure. Jugular venous pressure was not noted to be raised. His body mass index was 29 kg/m^2^. Blood tests showed a cholestatic picture, including raised bilirubin [72 μmol/L (normal range 0–21 μmol/L)] and alkaline phosphatase [ALP; 219 IU/L (normal range 30–130 IU/L)] with a normal alanine transferase [31 IU/L normal range (10–49 IU/L)].^9^ Albumin was normal at 40 g/L (normal range 35–50 g/L) and platelets mildly low at 107 × 10^9^/L (normal range 140–400 × 10^9^/L). B-Type natriuretic peptide (BNP) levels were not performed on initial presentation. The patient was admitted, and ultrasound liver demonstrated abnormal biphasic flow pattern in the portal vein, suggesting portal hypertension, with prominent hepatic veins and normal liver texture.

He was subsequently referred to gastroenterology as an outpatient, and a non-invasive liver screen was undertaken. This included alpha-fetoprotein; human chorionic gonadotropin; viral screening for hepatitis, Epstein–Barr virus, human immunodeficiency virus, and cytomegalovirus; cancer antigen 19-9; and ferritin, which were all negative. A liver biopsy reported features in keeping with congestive hepatopathy,^10^ with dilated sinusoids, no steatosis and bridging fibrosis, as well as bile duct reaction,^10^ with absent copper-associated protein and alpha-1 antitrypsin globules.

Three months following the liver biopsy, outpatient transthoracic echocardiogram (TTE) was performed. This demonstrated severe TR and a dilated RV with reduced systolic function with bi-atrial dilatation and mildly reduced LV systolic function. The inferior vena cava (IVC) was dilated at 3.1 cm and fixed in inspiration. This was also the first TTE performed following the downgrade of the ICD from dual to single chamber. When compared to surveillance TTEs performed previously, there had been a progression of previously reported TR from moderate to severe, and new RV dilatation and reduced function. For example, the TTE 1 year post-implantation of the original dual-chamber ICD had a new finding of moderate TR, with features of the known HCM and no outflow obstruction; normal left ventricle (LV size and function but with severe asymmetric LV hypertrophy); mid to apical septal thickening, septal thickness at the end of diastole 2.4 cm, LV internal diameter at the end of diastole of 2.4 cm, and LV posterior wall end diastole of 1.3 cm; mild mitral regurgitation with no systolic anterior mitral motion; bi-atrial dilatation; normal RV size and function; and no aortic or pulmonary valve disease. With the new finding of severe TR and RV dysfunction and liver biopsy in keeping with congestive hepatopathy, it was concluded that the severe TR was secondary to the RV ICD lead.

**Figure 1:**
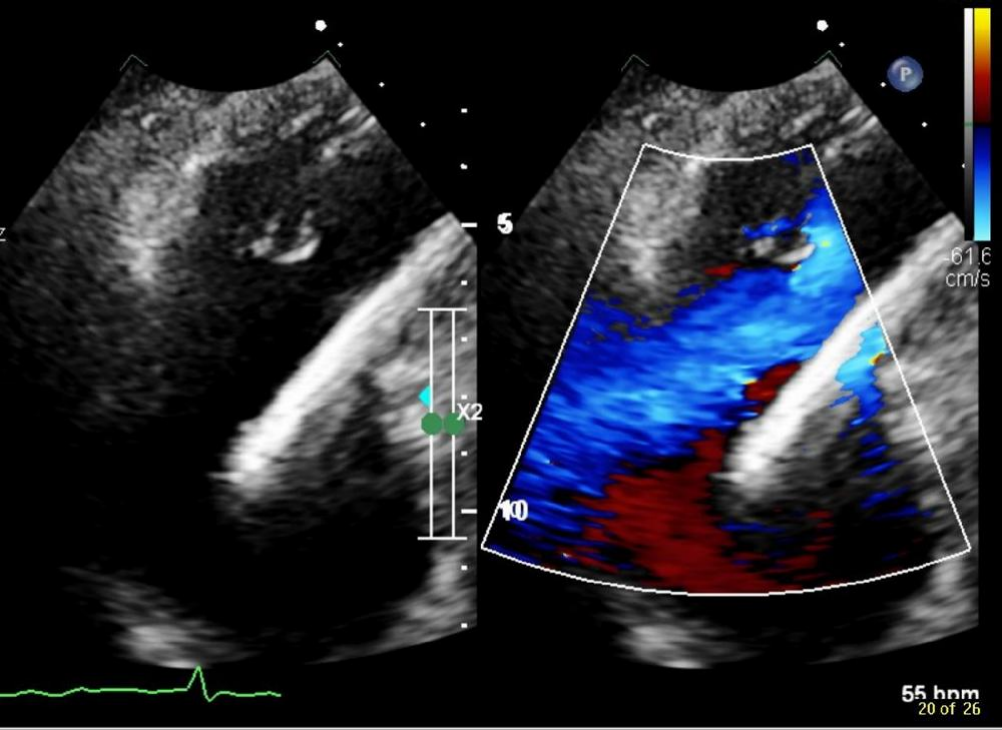
Apical four-chamber view, zoom on the tricuspid valve. Right ventricular pacing lead splinting open the septal leaflet causing a large coaptation gap.

**Figure 2:**
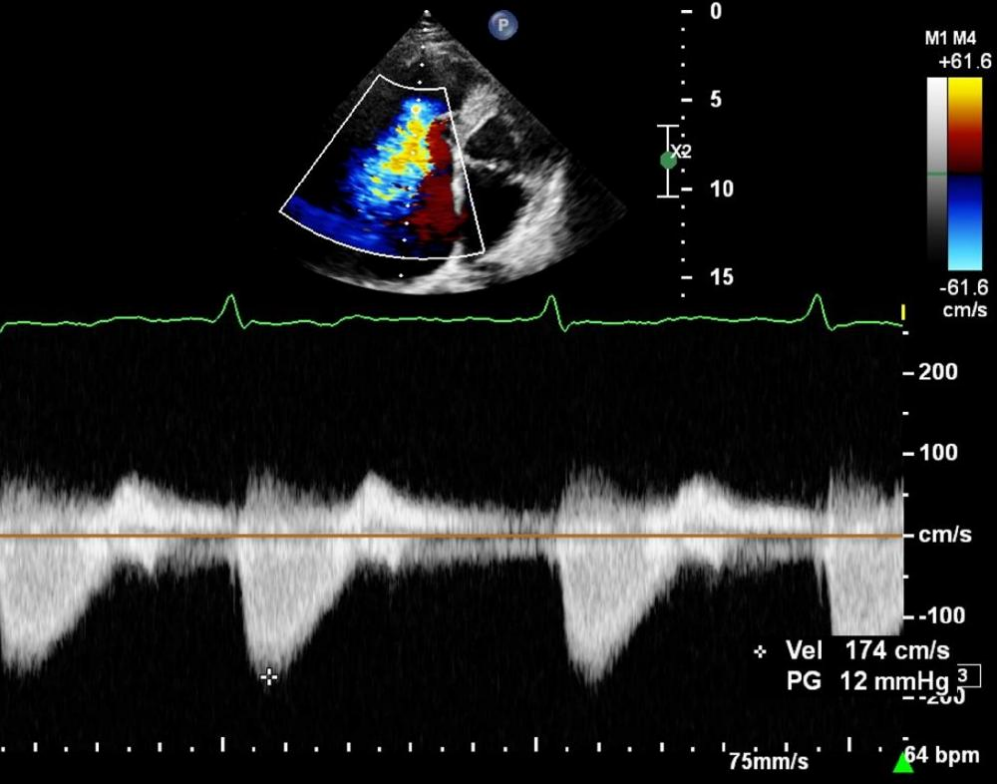
Apical four-chamber view, continuous wave Doppler through the tricuspid valve. Rapid equalization of pressure in an early peaking dense severe tricuspid regurgitation jet.

Nine months after the initial presentation with jaundice, on review in cardiology clinic, the patient had developed mild ankle oedema and a raised JVP, with a BNP of 653 ng/L. Right heart catheterization demonstrated normal pulmonary pressures (pulmonary artery pressure: 24/11 mmHg; mean pulmonary artery wedge pressure: 12 mmHg), with left heart disease excluded as cardiac output fell within normal limits. The study was highly suggestive of severe TR as the right atrial pressure waveform demonstrated a V-wave of 24 mmHg. To determine the mechanism of TR, transoesophageal echocardiography (TOE) was undertaken and suggested two mechanisms: firstly, the ICD lead was causing impingement on the septal tricuspid leaflet resulting in mal-coaptation (TTE images at the time are shown in *Figures 1* and *2* and Supplementary material online, *Videos S1* and *S2*) and secondarily by annular dilatation. The downgrade procedure to single-chamber ICD may have impaired the septal leaflet function; however, re-review of the TTE from 2 years post-implantation showed a similar position of the lead. For this reason, it can be presumed that over time, with concomitant AF, the tricuspid annulus dilated, aggravating TR and eventually leading to congestive hepatopathy.

Following diagnosis, tricuspid valve annuloplasty and ICD explantation were planned; however, during surgery (after the lead was removed), the septal leaflet had significant damage and subsequently the patient underwent tricuspid valve replacement using a 33 mm Perimount Magna valve. Intravenous heparin was used as peri-operative anti-coagulation. Post-operative medication changes included co-amoxiclav, aspirin 75 mg once daily, furosemide 20 mg twice daily, paracetamol, lactulose, and lansoprazole 30 mg once daily. Ultrasound liver post-procedure demonstrated a return of normal antegrade hepatic flow, and ALP had normalized, with bilirubin stable at 20–23 μmol/L. Transthoracic echocardiogram 2 years post-procedure showed the replaced tricuspid valve *in situ*, well seated, with a mean gradient of 3 mmHg, mild TR, and intermediate likelihood of pulmonary hypertension. The IVC remains dilated (2.7 cm), but now collapsing >50% on inspiration, and, although RV function overall appeared impaired, this was deemed to be consistent with previous cardiac surgery. There was a new finding of mildly impaired LV function with no other significant changes in echo parameters. At this point, diuretics started post-operatively were discontinued, and at 4-year follow-up, they have not been resumed. Cardiac biopsy taken intra-operatively confirmed HCM. Whilst awaiting a follow-up to discuss re-implantation of ICD, the patient presented to the emergency department with syncope. Electrocardiogram on admission showed AF with narrow complex and no evidence of a bradyarrhythmia. A S-ICD was implanted during the admission. This was chosen over a conventional transvenous ICD (TV-ICD) to prevent future damage to the newly replaced tricuspid valve. At 4 years post-procedure, the patient in our case continues to undergo annual cardiology and gastroenterology follow-up. He reports improved exercise tolerance and has not developed symptoms or signs of heart failure.

## Discussion

This case highlights a rare presentation (isolated cholestatic jaundice) of a well-recognized ICD complication (TR). To the best of our knowledge, isolated presentation of liver dysfunction from TR post-ICD implantation has not been reported previously. This builds on two previous cases in which liver dysfunction was associated with tricuspid pathology (tricuspid stenosis and TR) associated with an implanted cardiac device^11,12^; both patients however had more overt signs of right heart failure. Our case highlights that signs and symptoms of decompensated heart failure cannot always be relied upon to identify congestive hepatopathy.

In our case, an S-ICD was deemed an appropriate device to implant post-tricuspid valve replacement as this avoided a RV lead. Subcutaneous ICD trials have shown successful conversion of all ventricular arrhythmias, with registry data finding an acceptable inappropriate shock rate of 8.1% at 1 year.^13,14^ A pooled analysis of comparison studies of S-ICD and TV-ICD found no significant difference between appropriate and inappropriate shocks, whilst device and lead-related complications favoured S-ICD.^15^ Should the patient require a pacemaker in the future, there remains the option to combine an S-ICD with a leadless cardiac pacemaker.^16^

It is important to note that risk factors for cardiac implantable electronic device-related TR are well described and should be minimized, for example, by optimizing rate and/or rhythm control of AF, using the least possible number of leads, aiming for TV passage angle of −15° to +15°, placement technique (prolapsing vs. direct crossing), and placing the lead centrally using 3D TTE.^4,17^

In conclusion, the learning points of this case were as follows: liver dysfunction associated with severe TR is a potential complication of ICD implantation with an RV lead; TR due to transvenous RV lead may present with jaundice secondary to isolated congestive liver dysfunction; tricuspid annuloplasty or TV replacement, with explantation of ICD, can be a successful therapeutic intervention; finally, in suspected patients, due to the treatable nature of this complication, timely investigation (TOE and right heart catheterization) and subsequent treatment are important.

## Supplementary Material

ytae600_Supplementary_Data

## Data Availability

The data underlying this article will be shared on reasonable request to the corresponding author.

## Lead author biography

Dr Suzannah Fleming is an internal medicine trainee in the East Midlands, UK, with an interest in heart failure, imaging, and cardiac devices. She is currently completing her medical training with a vision to enter cardiology specialty training.

## Notes

**Consent:** We can confirm that we have consent in line with COPE guidance from the patient to publish and share this information.

**Funding:** J.G. was supported by the Wellcome Trust Leicestershire Healthcare Inequalities Improvement Doctoral Training Programme (223512/Z/21/Z). Other authors declare no source of funding.
